# Thoracic Erector Spinae Plane (T-ESP) Block Together With Intertransverse Process (ITP) Block for Laparoscopic Abdominal Surgery: A Case Report

**DOI:** 10.7759/cureus.52711

**Published:** 2024-01-22

**Authors:** Carmine Pullano, Francesco Marrone, Saverio Paventi, Lorenzo Forasassi, Roberto Starnari

**Affiliations:** 1 Anesthesia, Villa Pia Clinic, Rome, ITA; 2 Anesthesiology and Critical Care, Santo Spirito Hospital, Rome, ITA; 3 Anesthesiology, Villa Donatello Clinic, Florence, ITA; 4 Anesthesiology, Istituto Nazionale di Ricovero e Cura per Anziani (INRCA), Ancona, ITA

**Keywords:** laparoscopic surgery, superior costotransverse ligament, thoracic paravertebral space block, truncal blocks, intertransverse process block, thoracic erector spinae block

## Abstract

Laparoscopy has become a milestone with reduced surgical stress and postoperative pain. Evidence promotes erector spinae block for laparoscopic abdominal surgery, in particular for cholecystectomy. The thoracic paravertebral space block is the administration of local anesthetic into a wedge-shaped space on the antero-lateral thoracic spine and provides abdominal analgesia. We hypothesized that a combination of two paravertebral by proxy blocks (erector spinae and intertransverse process (ITP)) with multi-dermatomeric coverage and visceral pain control, with evidence for intra- and postoperative analgesia in thoracic and abdominal surgeries, may be a surgical anesthesia option for laparoscopy. A 42-year-old patient with gastroesophageal reflux disease (GERD) was scheduled for a laparoscopic Nissen fundoplication. He was 173 cm in height and weighed 90 kg (BMI 30 kg.m^-2^) and was classified in the American Society of Anesthesiologists Physical Status Classification System (ASA-PS) as 2. He had a history of difficult airway and refused general anesthesia. With the patient's informed written consent, we performed a bilateral thoracic erector spinae plane (T-ESP)/ITP blocks at the T4-8 level. Surgery was performed with the patient spontaneously breathing under sedation without complications. Hence, the combination of ESP-ITP blocks was a good anesthesia option for the planned surgery without side effects and optimal postoperative pain control.

## Introduction

Laparoscopic abdominal surgery has become a mainstay of modern surgery with reduced surgical stress and postoperative pain. Evidence promotes thoracic erector spinae (T-ESP) block (T7-9) for laparoscopic abdominal surgery in particular from studies investigating pain following laparoscopic cholecystectomy (LC) [1,2]. The ESP block first described by Forero et al. in 2016 [3] has been shown to be useful in many surgeries. Despite this mounting clinical evidence, the ESP block mechanism of action continues to be debated although the potential spread of local anesthetic (LA) into the paravertebral and epidural space and its distribution in the ESP plane and intercostal space are demonstrated in several anatomical studies [4]. The intertransverse process (ITP), according to an American Society of Regional Anesthesia and Pain Medicine (ASRA)/European Society of Regional Anaesthesia and Pain Therapy (ESRA) consensus on standardizing nomenclature [5], is currently used to indicate the midpoint transverse process-to-pleura block [6] and costotransverse foramen block [7], two paravertebral by proxy blocks [8] in which the mechanism of action is related to the potential spread of LA in the paravertebral space through the superior costotransverse ligament (SCTL). The thoracic paravertebral space (TPVS) block, as described in the 20th century, is the administration of LA into a wedge-shaped space on the antero-lateral thoracic spine (T6-9) and provides abdominal analgesia [9,10]. Pre-induction TPVS block resulted in improved analgesia for 24 h following LC, along with a significant reduction in perioperative opioid consumption and opioid-related side effects [11], and pre-incisional TPVS block conferred a significant preemptive visceral analgesic effect in patients undergoing LC significantly reducing the amount of postoperative opioid consumption [12]. We hypothesized that a combination of two truncal blocks, in particular two paravertebral by proxy blocks such as the ESP block and ITP block, with their mechanism of action related to the potential spread of LA into the paravertebral space, with their multi-dermatomeric thoracic and abdominal coverage, potential visceral pain control, and positive evidence for postoperative analgesia in abdominal surgery, may be a surgical anesthesia option for abdominal laparoscopic surgery without general anesthesia. Here, we present a case using bilateral ESP and ITP blocks at the T4-8 level in a patient undergoing laparoscopic Nissen fundoplication.

## Case presentation

**Figure 1:**
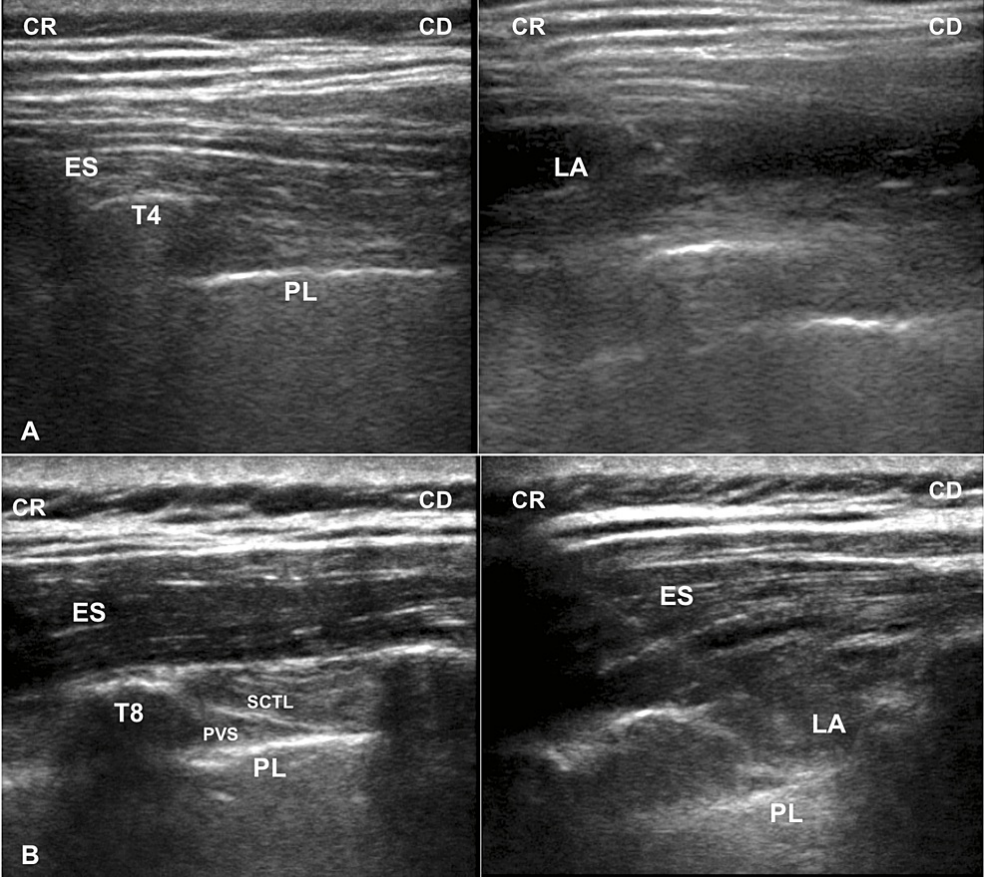
T-ESP block and ITP block A. T-ESP block B. ITP block is a paraspinal thoracic truncal block technique, where the LA is injected into the thoracic intertransverse tissue complex posterior to the SCTL T-ESP: thoracic erector spinae plane; ITP: intertransverse process; LA: local anesthetic; SCTL: superior costotransverse ligament; CR: cranial; CD: caudal; ES: erector spinae muscle; PL: pleura; PVS: paravertebral space

A written informed consent was obtained from the patient before the publication of this case report. A 42-year-old patient with gastroesophageal reflux disease (GERD) was scheduled for a laparoscopic Nissen fundoplication. He was 173 cm in height and weighed 90 kg (BMI 30 kg.m^-2^) and was classified in the American Society of Anesthesiologists Physical Status Classification System (ASA-PS) as 2. He had a history of difficult airway. After discussion with the patient who refused general anesthesia, we planned to perform the surgery without it; however, general anesthesia was proposed to the patient as a rescue strategy with an appropriate assistance for the anticipated difficult airway. With the patient's informed written consent, we performed bilateral T-ESP and ITP blocks. The patient was in prone position; then we located the T6 spinous process, and aseptically, with an ultrasound guide (Edge II, Fujifilm Sonosite™, Bothell, Washington, United States), in paramedian sagittal orientation, approximately 2 cm away from the midline to visualize the transverse process, we performed T4 ESP block; after that, we moved the 100 mm echogenic needle (Ultraplex™, B. Braun, Melsungen, Germany) caudally towards the T8 paravertebral space. The tip reached the retro-SCTL space between the intertransverse ligament and SCTL (Figure 1). We used in total a volume of 100 mL: 30 mL for ESP and 20 mL for ITP on each side. LA was ropivacaine 0.18%+dexamethasone 6 mg+dexmedetomidine 80 mcg. Intravenous midazolam 2 mg was administered before blockades and vital parameters were monitored. During LA injection, no adverse effects were noticed. Pneumothorax was excluded by lung ultrasound (LUS). Thirty-five minutes after, blocks were ascertained by pin-prick/cold test anesthesia in T3-T10 dermatomes and an hypoesthesia area up to C5. Supplemental oxygen was given by nasal cannula (3 L.min^-1^), and capnometry was used to have a reliable insight into ventilation, as the patient spontaneously breathing during laparoscopy. Ten minutes after the induction of pneumoperitoneum, the patient complained of shoulder pain. Intravenous dexmedetomidine 0.6 mcg.Kg^-1^.h^-1^ was started, and intravenous ketamine 25 mg (5 mg for five times) was added. Surgery lasted 90 minutes and was uneventful. No hypotension nor bradycardia registered. Before sedation emergence, paracetamol 1 g was administered and prescribed for postoperative analgesia. The Numeric Rating Scale (NRS) score at time 0 (emergence from sedation) was 2. In post-anesthesia care unit (PACU), the Ramsay score was 2-3. The patient was discharged from PACU after 40 minutes with an Aldrete score of 10. Two hours after surgery, we recorded an NRS score of 2. After six, 12, 24, and 36 hours, the NRS score was <4. The patient did not report postoperative nausea and vomiting. Paracetamol 3 g per day was given. The length of stay of the patient was three days.

## Discussion

To the best of our knowledge, this is the first case report in which in order to have an anesthesia option for abdominal laparoscopic surgery, a combination of two truncal blocks (in particular two paravertebral by proxy blocks such as ESP and ITP blocks) was provided. The ESP block was first described by Forero et al., and its mechanism of action is related to the potential spread of LA into the paravertebral space. A recent review by Lim et al. [13] including 29 anatomical studies found that T-ESP block has a consistent spread of injectate in the erector spinae compartment (100% of cases) spanning up to nine spinal levels and diffusion in the paravertebral space (57% of cases) with a mean of 3.5 spinal levels. The authors described also a time-related spread with 38% of paravertebral space diffusion at 30 minutes and 73% between 30 minutes and two hours. Moreover, only the group that received high volume (30 ml) had a paravertebral spread albeit limited to 2.7 spinal levels. ITP block, according an ASRA/ESRA consensus on standardizing nomenclature, is the new term indicating the midpoint transverse process-to-pleura block, with a mechanism of action probably related to the spread of LA in the paravertebral space. LA diffusion to the paravertebral space occurs through the SCTL. Anatomical and 3D micro-CT studies confirmed that the posterior border of the TPVS is not completely defined by SCTL, which in all examined cases (11 cadavers) always appeared micro-fissured. The retro-SCTL space, which contains the posterior branches of spinal nerves, is extensively connected to the costotransverse space, intervertebral foramen, and erector spinae compartment and to the TPVS through some slits in the SCTL [14]. The ITP block is applied on the posterior side of the TPVS (retro-SCTL space) [15] in a modality described as paravertebral block by proxy. A single-injection ITP block has shown to be non-inferior to multiple injections in terms of anesthetized dermatomes demonstrating an involvement to a variable extent up to four levels [16]. In a real-life clinical practice study, which reflects the clinical reality more accurately than autopsy studies, Chen et al. [17] found that compared with the ESP block, a single-injection ITP block yielded increased stability in the lateral and anterior chest wall block with improved anterior and intercostal spread although with a reduced cephalocaudal spread. The authors described an average diffusion segment of 10 for ESP and 4.5 for ITP. We hypothesized that the ITP block could enhance the ESP block, concentrating and improving its effects if the two blocks were combined. The ESP block with its potential spread to the paravertebral space (especially for high-volume injectates and after 30 minutes) and its consistent diffusion into the erector spinae compartment spanning up to 9-10 spinal levels extends coverage to more distant dermatomes in a synergistic effect with the ITP block. In Nissen laparoscopic fundoplication, trocars are supraumbilical, needing a T4-T10 coverage, also for gastric and esophageal surgical stimulations. Diaphragmatic peritoneum (C3-5) in our case had less anesthetic coverage even though ESP and ITP blocks overlapped. Last but not least, as in other loco-regional anesthesia contexts, LA and additives synergistically contributed to create a deeper sensory block and prolonged postoperative pain control, letting us to use high volumes of a diluted LA concentration avoiding risks of systemic toxicity. On the other hand, we have to note that surgery could not have been successful without a sedation regime with analgesic potential such as the combination of dexmedetomidine and ketamine. This represents a limit of our approach. Possible future research developments could involve the identification of increasingly lower but effective concentrations of LA with the use of adjuvants, aiming to safely administer larger volumes and achieve greater analgesic and anesthetic coverage in fascial blocks.

## Conclusions

In our experience, an ESP-ITP block combination was a good anesthesia option for the planned laparoscopic surgery of the patient without intraoperative side effects and optimal postoperative pain control. We are aware of the novelty and the concerns that such an approach to laparoscopy can generate, especially considering that the procedure was performed with the patient only sedated and breathing spontaneously. However, we are optimistic that a similar approach could be considered in other cases, proving to be effective and safe without side effects, without respiratory and hemodynamic complications, with optimal postoperative pain control, and with patient satisfaction. Further studies are needed to confirm our hypothesis.
